# 1643. Journey Mapping Penicillin Allergies in Children: Parental Experiences, Care Gaps, and Suggestions Offered by Parents to Address Unmet Needs

**DOI:** 10.1093/ofid/ofad500.1477

**Published:** 2023-11-27

**Authors:** Eileen J Carter, Kate Baron, Ellen Davies, Monika Pogorzelska-Maziarz, Elizabeth Monsees, Mary Lou Manning, Sherry Pagoto

**Affiliations:** University of Connecticut, Storrs, Connecticut; University of Connecticut School of Nursing, Storrs, Connecticut; The University of Adelaide, Adelaide, South Australia, Australia; Thomas Jefferson University, Philadelphia, Pennsylvania; Children's Mercy Kansas City, Kansas City, Missouri; Thomas Jefferson University College of Nursing, Philadephia, Pennsylvania; University of Connecticut School of Allied Health Sciences, Storrs, Connecticut

## Abstract

**Background:**

Penicillin allergies are commonly reported in children but rarely true and perpetuate the use of alternative antibiotics. Leading allergy societies recommend a proactive approach to penicillin allergy delabeling. We use patient journey mapping to identify opportunities to improve the delabeling of penicillin allergies in children before, during, and after a child’s initial reaction to penicillin.

**Figure:**
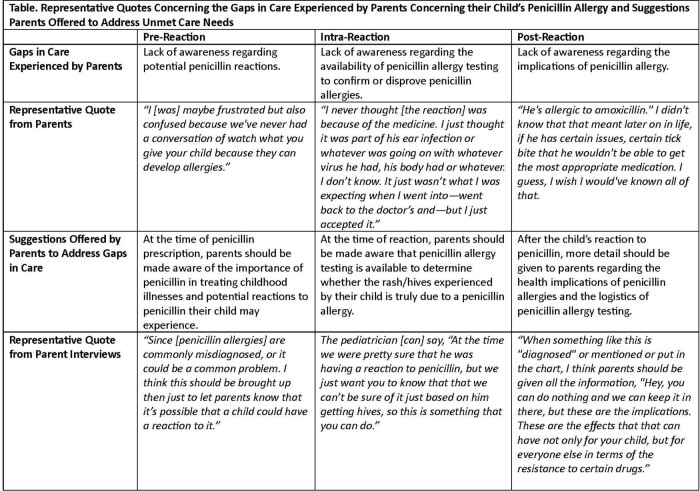

**Methods:**

Mapping was conducted via a secondary analysis of a qualitative study consisting of 18 interviews with parents recruited via social media reporting a penicillin allergy in their child. Two researchers coded interviews using a directed content analysis to capture parent’s experiences before, during, and after their child’s penicillin reaction. We describe the common experiences of parents ( >9 participants).

**Results:**

Pre-reaction, parents reported a lack of awareness regarding potential reactions children may experience when taking penicillin. Intra-reaction, parents reported their child’s provider suspected their child’s reaction was due to a penicillin allergy, wrote the child’s reaction in the medical record, and suggested or advised that penicillin be avoided in the future without mention of the availability of penicillin allergy testing. Post-reaction, parents remained unaware of the availability of penicillin allergy testing and uninformed of the implications of penicillin allergies on their child’s care. To better engage parents in penicillin allergy testing, participants suggested that providers inform parents of: 1) potential reactions their child may experience at the time of penicillin prescription (pre-reaction); 2) the availability of penicillin allergy testing to confirm the presence of true allergy (intra-reaction); and 3) penicillin allergy testing procedures and the benefits of penicillin allergy testing for their child’s health (post-reaction), see Table 1.

**Conclusion:**

Using patient journey mapping, we describe the common experiences of parents concerning their child’s penicillin allergy, gaps in care parents experienced, and suggestions offered by parents to address their unmet care needs. These findings may inform future efforts to improve the delabeling of penicillin allergies in children.

**Disclosures:**

**Sherry Pagoto, PhD**, Astellas Pharmaceuticals: Scientific Advisor|Weight Watchers, International,: Scientific Advisor

